# Advances in Procedural Techniques - Antegrade

**DOI:** 10.2174/1573403X10666140331142016

**Published:** 2014-05

**Authors:** William Wilson, James C. Spratt

**Affiliations:** 1 Edinburgh Heart Centre,; 2 Forth Valley Royal Hospital, Scotland

**Keywords:** Antegrade dissection re-entry, chronic total occlusion, knuckle wire, CrossBoss catheter, Stingray balloon.

## Abstract

There have been many technological advances in antegrade CTO PCI, but perhaps most importantly has been the evolution of the “hybrid’ approach where ideally there exists a seamless interplay of antegrade wiring, antegrade dissection re-entry and retrograde approaches as dictated by procedural factors. Antegrade wire escalation with intimal tracking remains the preferred initial strategy in short CTOs without proximal cap ambiguity. More complex CTOs, however, usually require either a retrograde or an antegrade dissection re-entry approach, or both. Antegrade dissection re-entry is well suited to long occlusions where there is a healthy distal vessel and limited “interventional” collaterals. Early use of a dissection re-entry strategy will increase success rates, reduce complications, and minimise radiation exposure, contrast use as well as procedural times. Antegrade dissection can be achieved with a knuckle wire technique or the CrossBoss catheter whilst re-entry will be achieved in the most reproducible and reliable fashion by the Stingray balloon/wire. It should be avoided where there is potential for loss of large side branches. It remains to be seen whether use of newer dissection re-entry strategies will be associated with lower restenosis rates compared with the more uncontrolled subintimal tracking strategies such as STAR and whether stent insertion in the subintimal space is associated with higher rates of late stent malapposition and stent thrombosis. It is to be hoped that the algorithms, which have been developed to guide CTO operators, allow for a better transfer of knowledge and skills to increase uptake and acceptance of CTO PCI as a whole.

## INTRODUCTION

Recent advances in the field of chronic total occlusion (CTO) percutaneous coronary intervention (PCI) have resulted in success rates of greater than 80% being reported in unselected and complex populations in centers with CTO PCI specific experience [1]. This compares favorably with historical data where success rates between 50 to 75% [2-5] were described. Whilst there have been developments in devices and techniques, a more fundamental change has been the evolution of a “hybrid” approach to procedural strategy, with a central tenet of using all feasible techniques (antegrade wiring, retrograde wiring, antegrade dissection re-entry or retrograde dissection re-entry) to achieve procedural success in the most efficient and expedient fashion. 

Recent studies have demonstrated the benefits of CTO PCI in alleviation of stable angina [6], as well as improvement in left ventricular function [7], quality of life [1] and in long-term survival [6]. Nonetheless, rates of percutaneous revascularization for CTOs remain low, with the presence of a CTO remaining both a strong predictor of referral for coronary bypass surgery (CABG) of medical therapy. Indeed, only 10-15% of CTOs are treated with PCI [8] and there is slow uptake of the newer techniques, including retrograde or antegrade dissection re-entry. Contributors to this low attempt rate include: skepticism regarding clinical benefit;operator inexperience, particularly with the newer techniques; a perceived greater risk of complications; and limitation of time and resources. 

This review will focus on advances in antegrade procedural strategy. 

## A. IMAGING

### i. Angiographic Assessment

#### A renewed Focus on Dual Injections

Detailed angiographic assessment is a critical part of the pre-procedural assessment. For successful antegrade CTO PCI, one must penetrate the proximal cap, traverse the occluded segment and distal cap before entering the distal true lumen. Although some of these segments may be defined by pre-procedure angiographic review, more information will be gleaned from dual injections (antegrade and contralateral) performed at the time of the CTO PCI [1]. Such injections, when performed properly on the appropriate magnification setting and without panning, allow identification of collateral supply as well as assessment of both occlusion length and the quality of the distal vessel. 

#### A Detailed Assessment of Collateral Filling Patterns

Definition of the presence and type of collateral supply is important not just to facilitate potential retrograde access but also to define any antegrade collateral contribution to distal flow, which may potentially be compromised during antegrade PCI attempts (either through antegrade dissection or deliberate branch occlusion with anchor balloons).

#### Angiographic Features Predicting Procedural Complexity

Identification of anatomic features of procedural complexity may help in negotiating these successfully. Recognized features include the presence of significant coronary calcium and vessel tortuosity [9]. Other factors, including the morphology of the proximal cap (blunt or tapered), the presence of bridging collaterals and length of occlusion, have less relevance with the continued evolution of procedural techniques. The J-CTO score provides an objective measure of case complexity, with 4 of the 5 parameters being defined angiographically [10]. 

### ii. Coronary CT

**Fig. (1):**
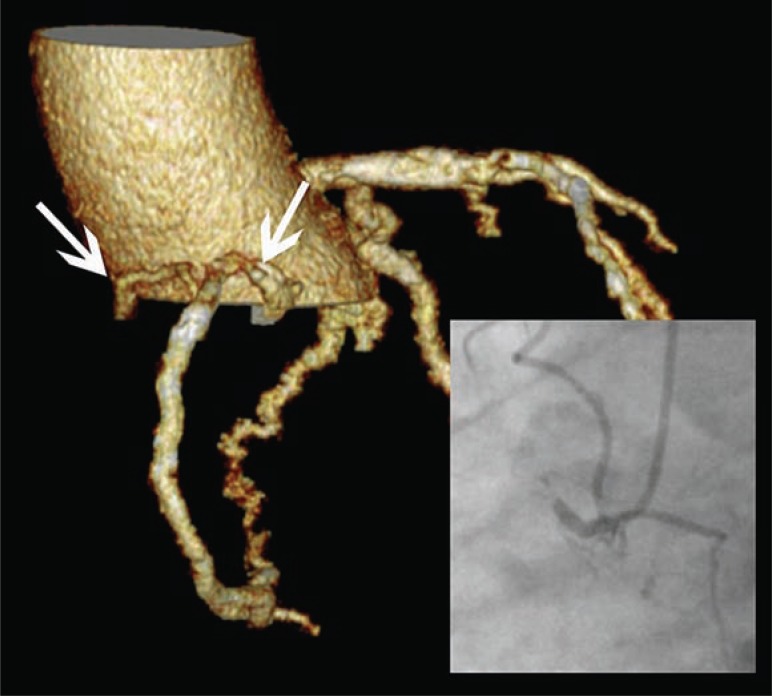
Use of co-registered cardiac CT to aid CTO PCI. Marked ambiguity existed in the proximal occluded segment with regard to course of artery and relation of atrial branches. CT reconstruction in the RAO 30 degree equivalent view proved useful in facilitating antegrade PCI by affording confidence with the direction of antegrade wiring and subsequent knuckle formation (initial cineangiogram in LAO projection).

The evolution of cardiovascular computed tomography angiography (CCTA) has proven useful in providing guidance for antegrade CTO PCI through better understanding of plaque morphology, the presence/degree of calcification and occlusion length [11]. Unlike cine angiography, CCTA can image the course of an occluded segment, and thereby resolve issues of anatomic ambiguity within occluded segments. CCTA can also provide useful information for ostial occlusions, anomalous coronary origins or insertions of occluded bypass grafts. Furthermore, new software systems that allow co-registration of the CCTA image with angiographic images can afford simultaneous appreciation of CCTA and live images. Co-registration may be particularly useful in antegrade dissection/subintimal tracking when side branches are present within the occluded segment as early identification of tracking into a side branch can be appreciated (Fig. **1**). The selected use of CCTA in patients with complex anatomy or with a previously failed attempt is currently preferred over systematic use due to concerns regarding added contrast load and radiation burden.

## B. EQUIPMENT

### B1. Guidewires 

An improved understanding of the histopathology of CTOs has led to development of new techniques and equipment for CTO-PCI. Autopsy analyses have revealed shorter duration CTOs to have collagen rich fibrous tissue at the proximal and distal ends of the occlusion. These “caps” are more rigid than the original lumen area forming the CTO body, which consists of “loose tissue segments” made up of organized thrombus, loose fibrous tissue and micro-channels (up to 250μm in diameter). By contrast, longer duration CTOs tend to contain harder intimal plaque and more dense calcium formations with less “loose tissue segments” and finer micro-channels [12, 13]. It has become clear from animal model studies [14] that micro-channels can be either extravascular (usually seen in the early phase of artery occlusion) or intravascular (which tend to develop in more mature CTOs). Sharply angulated connections exist between intravascular and extravascular micro-vessels in all phases of CTO development, whilst the intravascular micro-vessels became finer and more tortuous with time. Nonetheless, longitudinal continuity of the intravascular micro-channels extended to approximately 85% of the CTO length in the study by Munce *et al*. [14]. It is apparent that such loose tissue segments and micro-channels exist in both newer and more mature CTOs, thus may form a route for successful angioplasty and have provided impetus for developments in guidewire technology. 

**Fig. (2):**
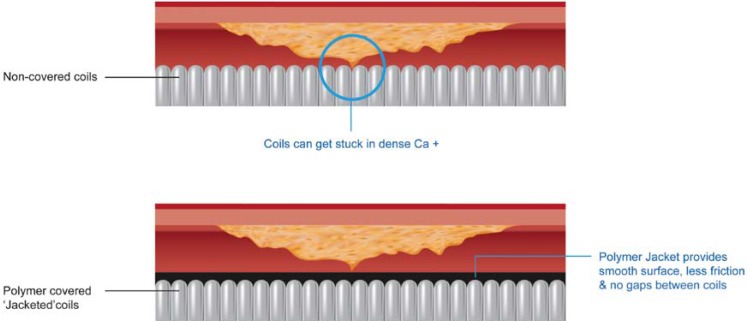
Graphic demonstrating mechanism for lubricity of jacketed wires.

Wire design improvements have included changes in core design, tapered tips, hydrophilic coatings and variable tip stiffness. Historically, non-coated and non-tapered wires with increasing tip stiffness have been used to drill through the CTO. As gleaned from histopathologic studies, intimal wiring can be via microchannels or loose tissue tracking, and tapered tip wires, whose tip approaches the size of such channels, have been developed for this purpose. Use of a tapered tip wire in a retrospective study of 182 patients in Japan was associated with an improvement in success rates from 65% to 81% (p=0.019) [15]. In this study, the tapered wires employed were CrossIt (Abbott Vascular, Santa Clara,) and Conquest (Asahi Intec, Nagoya, Japan), both of which do not have polymer jackets. Jacketed wires track with greater ease through these channels (see Fig. **2**) using a soft sliding technique and tapered low gram-force wires with tapered 0.009-inch tips have been developed including the Fielder XT (Asahi Intec).

**Fig. (3):**
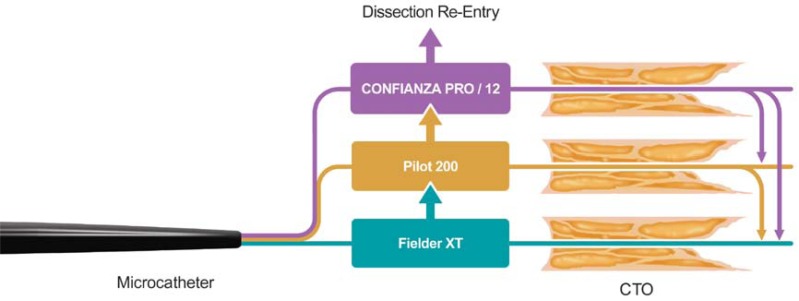
Antegrade wire algorithm.

If wire passage is unsuccessful with a jacketed low gram-force wire, escalation is often attempted with a jacketed guidewire with higher penetration power (eg Pilot 200 (Abbott Vascular). This wire is well suited to long and complex lesions and performs well in highly tortuous segments with ambiguous courses. If passage through the proximal cap still fails, usually due to inability to cannulate a microchannel or penetrate a hard and calcified proximal cap, wires with increasing tip stiffness have been developed to allow successful penetration. A commonly employed approach is to “step up” directly to very high tip load wires (examples include Confianza Pro 12 (Asahi Intec), which is a high–gram force wire with a hydrophilic coating and a 0.009 inch tapered hydrophobic tip for tactile feedback, Progress 200T (Abbott Vascular) or ProVia 15 (Medtronic, Minneapolis, MN) to puncture the proximal cap. Once the proximal cap has been penetrated, one “steps down” to a low tip load wire to progress through the occluded segment (using a micro-catheter to facilitate wire exchanges whilst maintaining position). It is important to note that high-gram force wires are best reserved for situations when the vessel pathway and location target coronary segment are well understood (Fig. **3**).

**Fig. (4):**
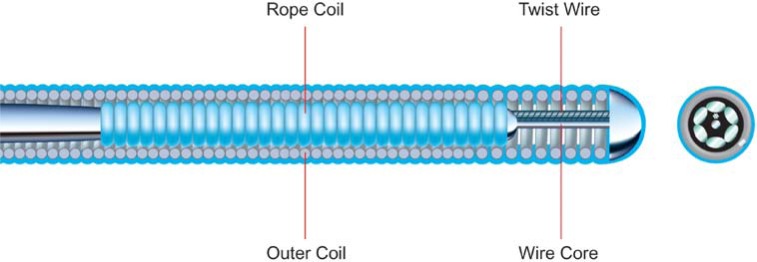
Sion wire.

A major advance in guidewire design has been the evolution of the Sion guidewire family (Asahi Intec), whose non kink one-piece core wire design makes it very torque responsive (Fig. **4**)– its utility in antegrade PCI lies in negotiating the segment proximal to the CTO and also distal to the CTO once the distal lumen has been entered. Its particular strength, however, is negotiating tortuous collaterals, as part of a retrograde procedure.

### B2. Support 

Increased support is a frequent requirement in CTO PCI, either for assistance with wire passage, penetration of the proximal cap, or delivery of a balloon through the occlusion once a guide-wire has successfully been directed to the distal vessel (which represents a mode of failure in up to 10% of CTO PCI [16]). New techniques and strategies have been developed for this purpose including micro-catheters, over-the-wire balloon catheters, the Corsair catheter, the Tornus catheter as well as more supportive guide catheter arrangements, such as mother-and-child systems.

### i. Micro-catheters 

Micro-catheters (small diameter over-the-wire catheters with low crossing profiles) are now routinely used as part of antegrade CTO PCI procedures for the purpose of:

1. Maintaining wire position between wire exchanges (allows for: 1. easy use of a workhorse wire to deliver to the proximal cap then rapid exchange for a CTO specific wire which may otherwise be traumatic to the proximal vessel 2. rapid escalation / de-escalation between different gram-force wires when required 3. changing the tip shape of the wire)
2. Improving wire torque response and provision of backup support to the coronary guidewire (allowing co-axial application of force by the wire even in tortuous vessels). By varying the distance between the end of the micro-catheter and the wire tip, microcatheters can serve to create a “dynamic wire tip load”: for example, a 12g wire tip load can be doubled by shortening its protrusion from the tip of a micro-catheter from 10mm to 6mm [17].
3. Penetrate the CTO once a wire is through. In contrast to most balloon catheters, micro-catheters are often designed with a soft, atraumatic, tapered tip allowing for better tracking through an occluded segment over the wire and for more co-axial force transmission.

Several micro-catheters are available currently and their specific characteristics are described in Table **1**. 

Other micro-catheters which are used less commonly incorporate distal tip angulation which can be useful in ostial CTOs or those with significant angulations in proximal vessel; examples include the Supercross catheter (Vascular solutions, Minneapolis, MN) with a pre-shaped distal tip to provide passive angulation, and the Venture catheter (St Jude Medical, St Paul, MN) which allows active angulation of the tip, controlled by a proximal tip deflection twist knob. 

An alternative to the micro-catheter is the over-the-wire angioplasty balloon, now available in diameters under 1.5mm. These are less expensive and can provide excellent wire support, but are generally less “trackable” than micro-catheters. In addition, balloon catheters tend to have a rigid distal tip, which can limit their ability to access angulated lesions and ironically reduce support. It is worth noting that balloons with a single middle radio-opaque marker band (usually those under 1.5mm diameter) are more trackable than those with two (proximal and distal) marker bands.

**Fig. (5):**
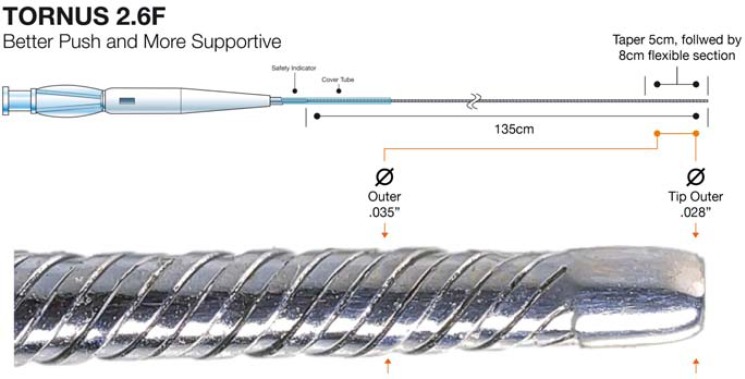
Tornus catheter.

The Tornus micro-catheter (Fig. **5**) (Asahi Intec) is a novel, over-the-wire, flexible, tapered micro-catheter designed for antegrade use and made from eight individual stainless steel wires, braided with a left-handed thread. It is designed to advance into a lesion over a coronary wire with a screw-like action (rotated into the lesion counterclockwise and withdrawn clockwise); it is a highly supportive microcatheter and can be used when distal wire position has been achieved but another micro-catheter or OTW angioplasty balloon will not cross a lesion, and is particularly useful in combination with rotablation [18]. Once across a lesion, the Tornus catheter will either allow balloon tracking through the track it has created (channel preparation) or else afford wire exchange, thus facilitating rotablation in heavily calcified lesions. The Tornus catheter has a porous design and as such cannot be flushed or aspirated. It is available in both 2.1Fr and 2.6Fr diameters, with the larger size being of most use in antegrade CTO procedures.

**Fig. (6):**
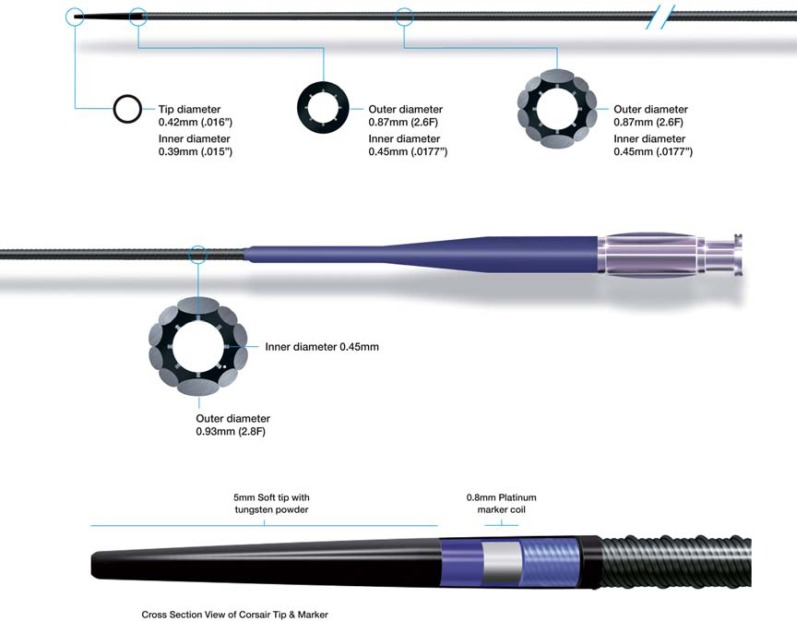
Corsair catheter.

The Corsair micro-catheter (Fig. **6**) (Asahi) is formed from tungsten braiding and 10 elliptical stainless steel braids, thereby combining the characteristics of the Tornus device and a micro-catheter. It has a tapered soft tip and a hydrophilic polymer coating for the distal 60cm, which facilitate tracking through tortuous segments as well as an inner polymer lumen for optimal wire control. It excels as a catheter for collateral channel crossing (usually retrograde) [19] but can be used in the antegrade direction for wire support/exchange as well as lesion crossing, with generally a more favorable crossing profile than other antegrade micro-catheters. 

### ii. Guide Selection 

Increased guide support can be achieved with larger diameter guides (up to 8 French via femoral artery), with more aggressive shapes or through deep intubation in the coronary tree. Larger diameter size guiding catheters offer additional benefits such as the ability to use balloon trapping or anchoring techniques, which can be challenging with guide sizes less than 7F. Many operators prefer to utilize the radial artery for access (either bi-radial or radial-femoral), to reduce morbidity associated with bleeding encountered with femoral access; the advent of sheathless guides has allowed operators to use guides with large internal lumens via the radial approach. 

### iii. “anchor” Balloon 

**Fig. (7):**
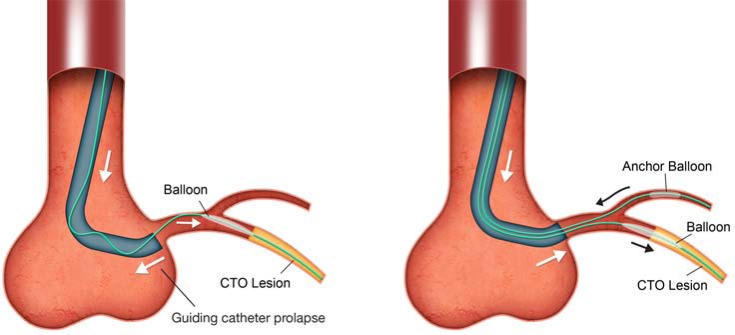
Anchor balloon.

Further support can be achieved with an anchor wire or anchor balloon technique (Fig. **7**) where balloon inflation on a separate wire in proximal side branches acts as an “anchor” [20]. The anchor balloon technique is particularly useful in RCA CTO PCIs, where proximal atrial or right ventricular branches can be utilized. Care must be taken to avoid ischaemia-related complications during the procedure. The balloon used as an anchor can be deflated and withdrawn into the guide at times of equipment exchange during the procedure where it can also serve a dual purpose as a “trap” balloon. 

### iv. Guideliner / Heartrail

An additional technique to enhance guide catheter support is the use of “mother-and-child” guide catheters, which provide extra support through the introduction of a smaller catheter (child) into the artery through the guide catheter (mother). Available systems include the Guideliner (Vascular Solutions) and the Heartrail [22] (Heartrail, Terumo, Japan). The Guideliner catheter has a rapid exchange design and is available in 3 sizes (6F, 7F, 8F). 

Functions of a mother-and-child guide include:

1. Provision of extra support through extension into the coronary artery and coaxial engagement
2. Reduction in contrast use and protection of stents during delivery to the lesion.
3. Guideliner-assisted reverse CART: when antegrade dissection has been performed as part of a reverse CART technique, the Guideliner catheter can also serve as a target for the retrograde guidewire.

Care must be taken to avoid trauma to stents at the proximal aspect of the Guideliner catheter as they are delivered through the guide. 

## C. TECHNIQUE - USE OF THE SUB-INTIMAL SPACE 

**Fig. (8):**
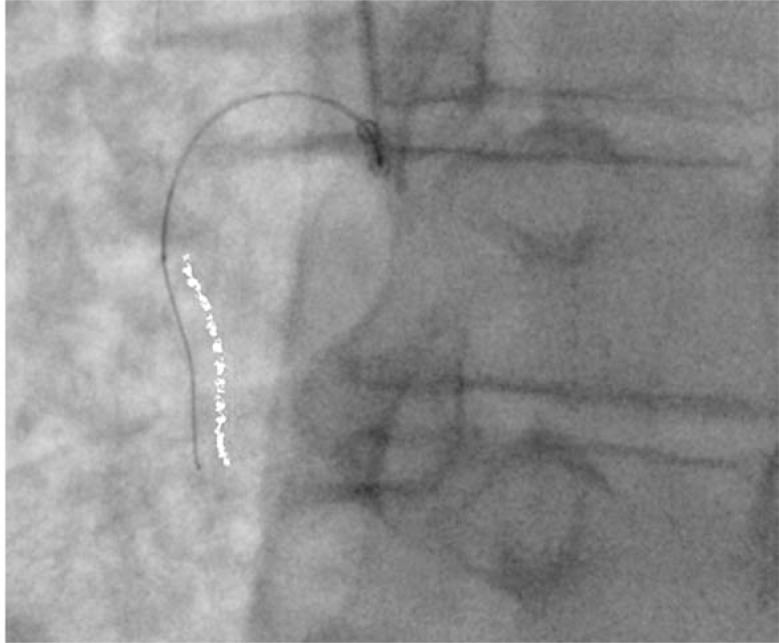
‘Sigmoid’ wire shape on angiography suggests subintimal wire passage.

Initial CTO PCI attempts were heavily focused on “luminal” wiring, with attempts made to avoid subintimal wire passage. This strategy of antegrade wiring with intimal plaque tracking remains valid in short CTOs (less than 20mm in length) with well defined proximal caps where it is associated with high rates of lumen-to-lumen wire passage [23]. In more complex anatomy however, its applicability has been limited by poor success rates, poor reproducibility and difficulties in teaching the technical intricacies required. Attempting to wire long CTO segments is also inefficient, with consequent long procedural times and high contrast/radiation use. Furthermore, inadvertent sub-intimal wire passage, as suggested by the “Sigmoid curve sign” [24] (see Fig. **8**), is common when attempting intimal plaque tracking, especially in calcified and tortuous lesions. Indeed, an IVUS based study [25] demonstrated sub-intimal passage occurred in 45% cases where a de novo CTO was crossed with a guide-wire. 

Previously in the setting of sub-intimal guidewire passage, the parallel wire or “see-saw” techniques were used. When utilizing the parallel wire technique, the wire that has tracked sub-intimally is left in place whilst a second, stiffer and more torqueable wire (eg Pilot 200 or Confianza Pro 12) is introduced via a micro-catheter (to avoid wire “wrap”) to facilitate steering into the true lumen [1]. Postulated benefits with this technique center on the first wire acting as a marker to minimize contrast load, whilst also occluding the entry site of the false lumen, as well as alteration of arterial geometry to reduce resistance for passage of the second wire. A modification of this technique is the “seesaw wiring method” where two guidewires in two micro-catheters are used alternately to increase the probability of finding the true lumen. In practice these techniques are unreliable and inefficient, as the first wire rarely occludes entry to the subintimal space, and it is difficult to avoid passage of the second wire into the same space as the first. Repeated tracking to the sub-intimal space can be explained by the fact that this space is weak longitudinally and offers less resistance than the intimal plaque located between the true lumen and the subintima. These techniques can also cause collapse of the distal true lumen through expansion of the sub-intimal space, which can hinder success rates offers less. Realistically, once a wire has entered the sub-intimal space, it is difficult to re-enter the true lumen without dedicated re-entry techniques. 

**Fig. (9):**
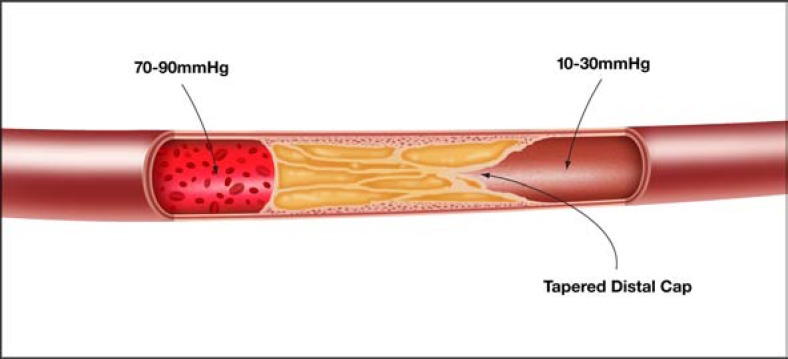
Proximal and distal caps.

Alternative strategies have therefore evolved, in particular to manage long and complex CTOs where antegrade wiring is associated with very long procedure times and invariable sub-intimal passage of the guide-wire. The retrograde approach, utilizing septal and epicardial collaterals, is attractive in the presence of an ambiguous proximal cap with a poor distal target and appropriate “interventional” collaterals (collaterals which are amenable to wiring). The distal fibrous cap is often tapered usually less rigid when compared to that of the proximal cap (Fig. **9**) [26] and consequently easier to penetrate. Sub-intimal passage is a preplanned event in the majority of retrograde PCI and it is relatively straightforward to join antegrade and retrograde sub-intimal spaces using a retrograde dissection re-entry technique, which capitalizes on the principle that the sub-intimal space is not only easily dissected in a longitudinal fashion but also in a transverse plane [24].

**Fig. (10):**
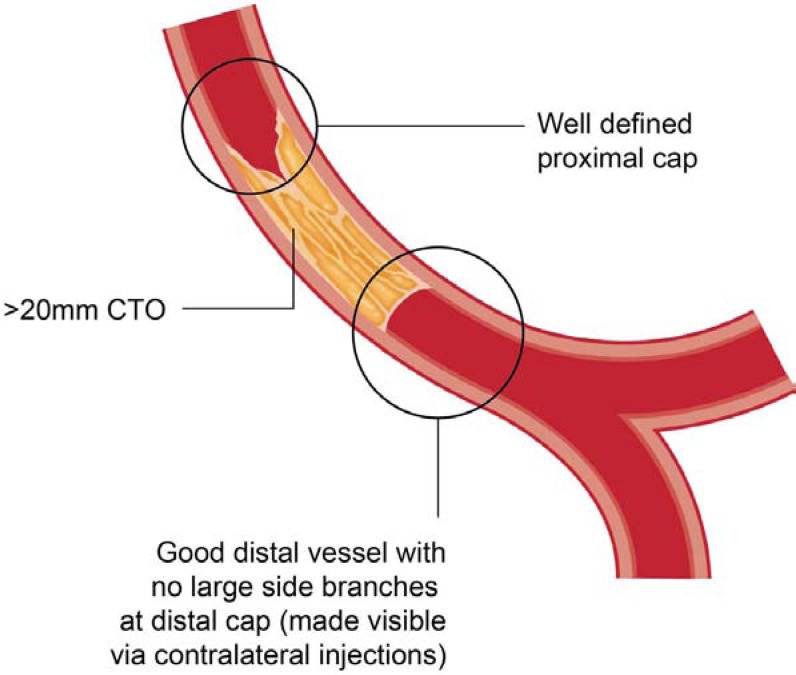
CTO anatomy suited to antegrade dissection re-entry strategy.

A subset of anatomy corresponding to about 20-30% of cases remain, however, where both direct antegrade intimal wiring and retrograde PCI are ineffective; in particular, long occlusions (>20mm) with poor “interventional” collaterals and a relatively disease free distal vessel. It is in these cases where a dissection re-entry strategy is useful (Fig. **10**). The concept of dissection re-entry with deliberate prospective dissection in the sub-intimal plane (within the vessel architecture, but outside the “true” lumen) for the length of an occluded segment with strategic re-entry after the distal cap into the distal lumen has developed to meet this need. 

It should therefore be evident that the choice of strategy should be dictated by the anatomy of the CTO, i.e. anatomy dictates strategy.

## DISSECTION RE-ENTRY

### 1. Historical Background

Sub-intimal angioplasty originated in the peripheral circulation, having been first described in 1989 [27] in percutaneous intervention for femoropopliteal occlusions. Despite this first description over 20 years ago, it has only recently achieved widespread clinical acceptance and has been extended in that arena to the distal crural vessels [28]. 

### 2. Rationale

**Fig. (11):**
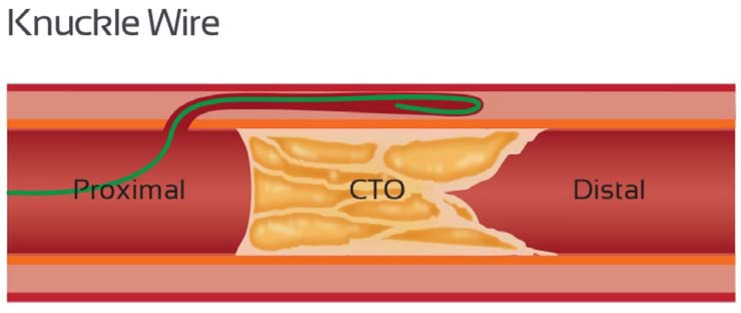
“Knuckle” wire.

The aim of sub-intimal angioplasty in this setting is to create a channel between the intima and media by means of intentional dissection, then to perform angioplasty on this channel to enlarge it and maintain its patency. This strategy is based on the principle of differential resistance between tissue planes and capitalizes on the relative longitudinal weakness of the sub-intimal plane, which allows rapid tracking to the distal lumen. Dissection is usually achieved with a looped polymer-coated wire (“knuckle”) (Fig. **11**), which can be advanced safely and rapidly within the sub-intimal space, usually with the leading end being the junction between the stiff and floppy part of the wire. This safe method capitalizes on the distensibilty of the adventitia; that is, blunt force can be easily applied within the vessel wall as it is delivered to the adventitia over a large area – causing blunt dissection, whereas stiff guidewire tips deliver significant force to a focal area with a high risk of perforation. In addition, the knuckled wire is less likely to find and perforate side branches. The primary limitation with this technique in peripheral artery CTO procedures has been failure to re-enter the distal true lumen after sub-intimal crossing of the occlusion (success rate estimated to be approximately 20% with a guidewire alone). Dedicated devices have therefore been developed to facilitate re-entry in peripheral arteries, including the Outback catheter (Cordis, Bridgewater, NJ) and the IVUS guided Pioneer catheter (Medtronic), which have dramatically improved the rates of successful re-entry (91% in one study [29]).

A similar principle has been applied to the coronary circulation, in particular for occlusions with a well defined proximal cap that are longer than 20mm with a reasonable distal vessel and no large side branches at the distal cap of the occlusion (Fig. **10**). The presence or absence of bridging collaterals, tortuosity or calcification does not impact on the decision algorithm. Strategies initially employed for this purpose included the STAR technique and its modifications. Current best practice would dictate that antegrade dissection is achieved with the CrossBoss catheter (Boston Scientific, Natuck, MA), whilst re-entry can be achieved with a Stingray balloon and wire (Boston Scientific).

### 3. STAR Technique and Modifications

#### 3.1. STAR

Within the coronary circulation, antegrade dissection re-entry use was first described as part of the STAR (sub-intimal tracking and re-entry) technique [30]. This involves the use of a knuckled guidewire advanced beyond the occluded segment into the sub-intimal space. This looped wire is then forcefully advanced to facilitate re-entry. This re-entry, however, usually depends on meeting resistance at a distal side branch or angulation and is therefore uncontrolled and unpredictable. Furthermore, it often necessitates creation of a long sub-intimal tract with the loss of major side branches and consequent poor distal run off. Re-creation of side branches can be attempted with “refenestration” techniques with jacketed wires to achieve reasonable initial angiographic results. This technique has, however, been plagued by both very high restenosis and early occlusion rates, compromising long-term patency [30]. It should, therefore, be reserved as a bail-out method and avoided in the left anterior descending artery due to risk of septal and diagonal branch compromise. 

#### 3.2. “Carlino” Modification

A modification of the STAR technique is the contrast enhanced STAR technique described by Carlino *et al.* [31] where contrast injection via a micro-catheter is used to create and delineate the dissection plane, which then can be wired using a soft wire. Although high rates of angiographic success have been described using this technique, it is also associated with relatively high rates of perforation and restenosis (45% restenosis rate at angiographic follow up at 7 months) [31]. As a technique, it tends to be of most use in relatively straight sections of occlusion, such as the vertical portion of the RCA. 

#### 3.3. Mini-STAR

Another modification is the mini-STAR technique, where a different wire shape is employed and re-entry is attempted as proximally as possible. This technique employs newer wires with a soft tip load and a hybrid coating, such as the Fielder FC or XT (Asahi Intecc). The wire tip is modified in a different way to the STAR technique (where a “J” loop configuration is made). The tip created has a small first curve (45-50 degrees 1-2mm proximal to the tip) in addition to a second curve (15-20 degrees 3-5mm from the tip). Via a microcatheter, intimal wiring (usually via microchannels) is attempted and if unsuccessful, forward pressure and creation of a small J-shaped loop allows sub-intimal tracking through the occlusion. Re-entry with the soft wire is expected to occur soon after the occlusion where, in theory, less tissue resistance is encountered; this technique was employed in 42 of 225 consecutive CTO procedures with a previous unsuccesful attempt, by Galassi *et al.* [32]. Recanalization was successful in 41 of 42 patients (98%) compared to 52% among patients in whom conventional crossing strategies (such as parallel wire, STAR, microchannel technique, IVUS-guidance, and anchor balloon) were used. Four patients treated with mini-STAR developed a perforation, with 1 case of tamponade requiring pericardiocentesis. No data exists on the long-term outcomes after use of the mini-STAR technique and further studies are required to corroborate the high success rates achieved in this study.

### 4. Collagenase

Another approach aimed at plaque modification is in development and involves local infusion of collagenase into CTOs, which appears to aid wire crossing [33]. Collagen is the predominant component of atherosclerotic plaque and local delivery of collagenase in animal models appears to have a differential effect on the matrix in occlusive plaque compared to the outer layers of the vessel wall. This has afforded higher rates of successful guidewire crossings in a rabbit femoral artery model (62% for collagenase treated arteries versus 29% for placebo treated arteries, p=0.028) without adverse effects on arterial structure, which might otherwise portend a higher perforation risk. A phase 1 study in 28 subjects with a CTO with a previous failed angioplasty attempt is currently underway to evaluate the safety and tolerability of this technique in humans.

### 5. Crossboss / Stingray

The CrossBoss/Stingray system (Boston Scientific) was designed in the face of a need for a CTO specific device that could facilitate expedient antegrade sub-intimal dissection without excessive vessel trauma, and allow controlled, predictable and reproducible re-entry to the distal lumen. 

#### 5.1. Antegrade Dissection – technique Specifics

Antegrade dissection can be reliably achieved through use of the knuckle wire technique or the CrossBoss catheter (Boston).

##### 5.1.1. Knuckle Wire

**Fig. (12):**
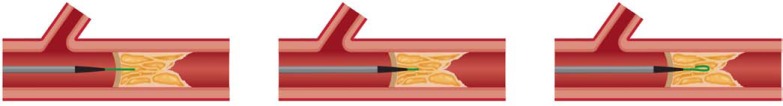
Penetration of proximal cap with stiff wire to allow introduction of microcatheter and soft wire to CTO body.

**Fig. (13):**
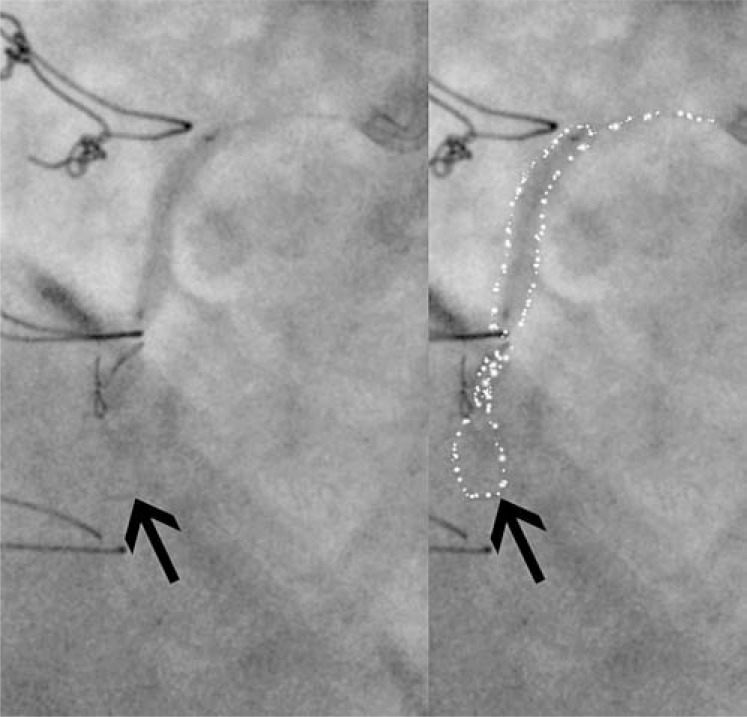
Knuckle formation with support of a proximal inflated balloon.

The knuckle wire has become an essential component of both antegrade and retrograde PCI, and is used in the same manner as in the peripheral circulation to negotiate occluded segments rapidly and safely with a low perforation risk. It is often used in conjunction with the CrossBoss catheter, rather than being a mutually exclusive technique. Commonly used wires for this purpose are polymer-jacketed wires such as the Fielder XT (Asahi) or Pilot 200 (Abbott Vascular) wires. To create a knuckle, the wire must loop on itself such that the leading edge is usually the junction between the stiff shaft and the floppy distal segment. The knuckle loop can be achieved by pre-shaping the wire tip as an “umbrella handle” and by applying forward pressure on the wire once it is within the CTO. It is not generally possible to enter the sub-intimal space without the proximal CTO cap first being penetrated with a stiff wire, or disrupted by other means, to then allow micro-catheter entry into the CTO body through which a polymer jacketed wire is introduced (Fig. **12**). To avoid knot formation when forming a knuckle, the wire should not be rotated in any way. In the presence of calcification and tortuosity, a large amount of force can be required, often utilizing measures previously discussed for support (large, deep seated guide with micro-catheter and anchor balloon for example). In cases of extreme resistance, a balloon (loaded on the knuckled wire) can be inflated in the vessel just proximal to the occlusion for maximal support (Fig. **13**).

It is preferable to keep the size of the knuckle as small as possible to minimize vessel trauma; loops formed with a Fielder XT wire tend to be smaller than those formed by the Pilot 200 wire, whilst the knuckle diameter can be controlled to some degree by adjusting the distance between the supporting micro-catheter and the knuckle tip. Ultimately, however, the knuckle size is determined by the diameter of the vessel, with the proximity of the micro-catheter to the knuckle tip determining the degree of force applied to create the dissection plane. The knuckle is primarily used to resolve anatomic ambiguity and in situations where extreme calcium / tortuosity does not permit tracking of a crossboss catheter. The principle of minimum vessel distortion should be applied and it is preferable that the majority of a subintimal tract be created by a CrossBoss catheter rather than a knuckle where possible.

##### 5.1.2. CrossBoss Catheter

**Fig. (14):**
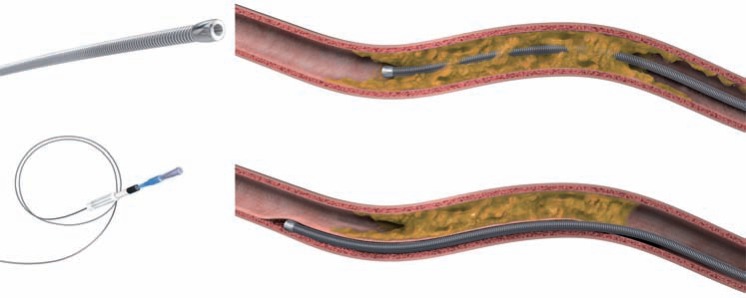
CrossBoss catheter: Upper – CrossBoss catheter passage “lumen to lumen”. Lower – CrossBoss subintimal passage.

The CrossBoss catheter (Boston Scientific) represents an additional method for antegrade dissection, which can be used alone or in a complimentary fashion to a knuckle wire. It is a 6 French compatible, stiff, metallic over-the-wire catheter (Fig. **14**) with a blunt, polished and atraumatic 3F (1mm) tip. The CrossBoss catheter can be delivered to the occluded segment in the same fashion as a micro-catheter, usually over a workhorse wire, but is then advanced ahead of the guidewire, and propagated forward using the “fast spin technique” (device rapidly rotated manually with a proximal rotating device) to allow crossing of the occlusion whilst remaining within the vessel architecture (either within the true lumen or by creating a controlled sub-intimal track). The blunt tip effectively acts as a “microknuckle” with the advantage of creating a smaller track than a knuckle does, thus minimising vessel trauma and increasing the chances of successful true lumen re-entry using the Stingray balloon and wire. It is worth noting that the CrossBoss passes directly into the distal true lumen in 20-30% cases [34], especially when used in LAD CTOs or CTOs due to in-stent restenosis [35]. In this scenario, the distal lumen can be wired simply by passing a guidewire through the CrossBoss lumen.

**Fig. (15):**
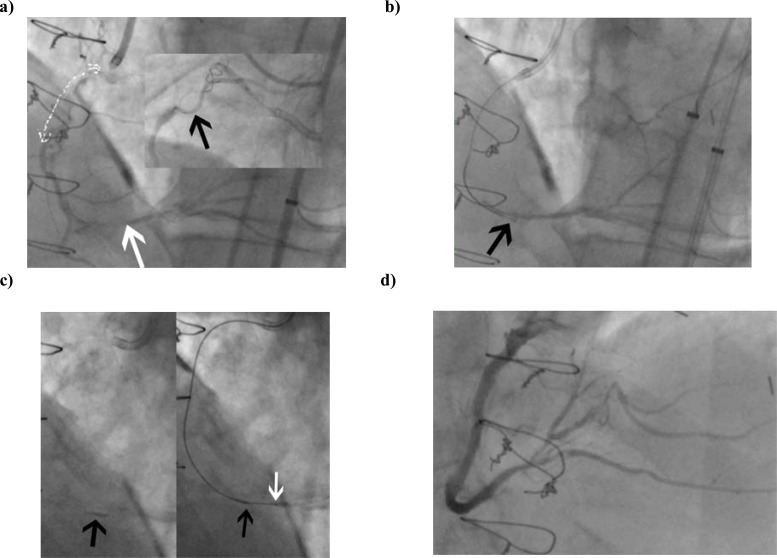
Case involving knuckle wire and CrossBoss with successful Stingray re-entry. Long proximal RCA CTO with moderate disease in distal vessel (white arrow). Inset = Balloon + knuckle formation to cross heavily calcified lesion (black arrow points to end of knuckle) b. CrossBoss used to extend sub-intimal track to distal “landing zone” (black arrow indicates CrossBoss tip) c. Stingray balloon (left) and wire re-entry (right – black arrow indicates wire exit site from balloon and white arrow indicates wire within true lumen) d. Final result.

The CrossBoss is particularly well suited to CTOs with a proximal tapered cap. In the setting of a blunt proximal cap, entry into the CTO body is necessary before introducing the CrossBoss in the same manner to that described for the knuckle wire technique. The CrossBoss catheter does not track as well in heavily calcified lesions; in such instances where the CrossBoss cannot “get started” or stalls, the knuckle wire technique may be preferred initially (Fig. **15**). Furthermore, the CrossBoss catheter can be used as a microcatheter to support knuckle wire formation. In the case described, a knuckle wire was used to cross the highly resistant and calcified occluded segment (with proximal balloon support), and the CrossBoss catheter introduced and tracked in the sub-intima distally to facilitate re-entry from a confined sub-intimal space (higher success rates with Stingray balloon and wire are achieved from smaller sub-intimal tracks). 

Perforation with CrossBoss use is uncommon unless it is passed into side-branches or if it follows a stiff wire, which has already exited. It is thus important that diligence is taken to recognize passage of the CrossBoss catheter into side branches. Assessing one’s position frequently in orthogonal views can identify this, but one must be wary of side branches running parallel to the main lumen. In the setting of repeated side-branch tracking, the catheter can be redirected using either a wire or a knuckle sufficiently large to track past the side branch. 

The CrossBoss is particularly well suited to crossing CTOs due to in-stent restenosis where success rates of >80% have been described [35] in association with short crossing times and low complication rates. It can rapidly negotiate occluded segments as it deflects off stent struts and is particularly suited to lesions with a tapered proximal cap without severe tortuosity. 

#### 5.2. Re-entry

Once the occluded segment has been crossed to allow equipment to be passed beyond the CTO to the sub-intimal space beside the distal true lumen, a re-entry strategy is required. The method of choice in this situation is a device-based method (Stingray balloon and guidewire (Boston), with wire-only based methods (LAST or IVUS guided) remaining bailout options only. 

##### 5.2.1. LAST (Limited Antegrade Subintimal Tracking)

The LAST technique [36] is similar to the mini-STAR technique but the knuckled wire is advanced to but not past the distal cap and, instead of using a soft, tapered tip looped wire (eg Fielder XT) for re-entry, a stiffer wire (Pilot 200 (Abbott Vascular) or Confianza Pro 12 (Asahi Intecc) with an acute distal bend is used via a micro-catheter in an attempt to re-engage the tissue within the distal body of the CTO to gain subsequent entry to the distal lumen. No data currently exists regarding the feasibility and outcomes of this technique but anecdotal reports are favorable.

##### 5.2.2. IVUS Guided Re-entry

IVUS can be used to differentiate a true lumen from a false lumen, and can therefore be employed to assist wire orientation to guide wire re-entry. After 1.5mm or 2mm balloon dilation in the sub-intimal space, an IVUS catheter is advanced into the space and is monitored to orient the second wire into the true lumen. The second wire should be a stiff, tapered wire delivered in a microcatheter, hence an 8-French guide is mandatory. This technique is demanding and requires spatial orientation with an ability to translate cross-sectional IVUS images into three dimensions in real time. This is complex and difficult to teach, which has limited its widespread uptake.

##### 5.2.3. Stingray Balloon

**Fig. (16):**
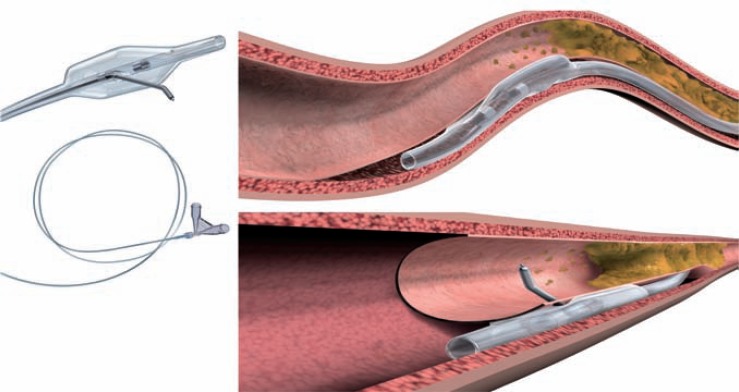
Stingray catheter: Upper panel- Inflated Stingray balloon in position in the subintimal space. Lower panel – Stingray guidewire entry into true lumen.

**Fig. (17):**
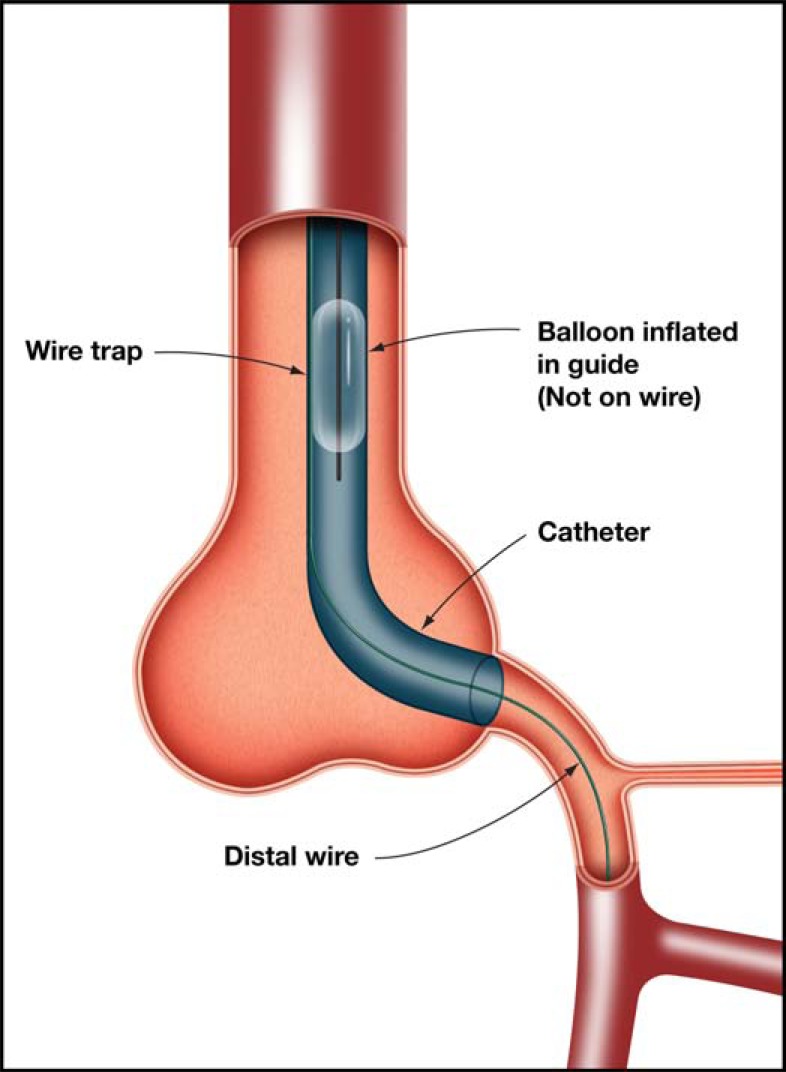
Use of a “trap” balloon.

The Stingray balloon and guidewire (Boston) has emerged as a dedicated system for controlled re-entry and is designed for use with the CrossBoss catheter (Boston). The Stingray balloon is a 1mm flat balloon that is 6F guide catheter compatible and has 3 exit ports connected to the same central 0.014-inch guidewire lumen. The distal exit port is used to deliver the device over a standard guidewire (akin to an OTW balloon) to the sub-intimal space at the site of planned re-entry. It is inflated to 4 atm and the shape of the balloon ensures it orientates itself adjacent to the lumen such that one of the remaining two exit ports (which are 180 degrees opposed to each other) will direct the Stingray wire towards the lumen. (Fig. **16**) Proper device preparation is important to afford visualization of the flat balloon. The ideal angiographic view for re-entry is one where the balloon is imaged tangentially rather than “en face” (Fig. **15c**). Imaging to define relationship to the true distal lumen is achieved by contralateral injection (antegrade injections should be avoided once antegrade dissection has occurred to avoid propagation of the dissection). The Stingray wire is a 0.014-inch high gram-force (12g) wire with a shallow preformed angulated distal tip and a short (1mm), very fine (0.04mm) distal probe that acts to grab tissue to facilitate penetration into the true lumen. For re-entry, the Stingray wire is advanced into the Stingray balloon and manipulated so that it exits the port facing the lumen. A characteristic “pop and release” sensation is experienced with entry into the distal lumen, corresponding to the resistance felt by the wire as it penetrates the vessel wall, followed by the absence of resistance as it enters the vessel lumen; at this point the Stingray wire can be advanced to the distal lumen if the artery is large and free of significant tortuosity. In general, however, a “stick and swap” approach is preferred to avoid dissection or luminal disruption with the stiff Stingray wire; having made one or more punctures, the Stingray wire is exchanged for a jacketed wire (Pilot 50 or 200 wire), which re-enters through the already created hole(s) and tracks more easily and safely to the distal vessel. At this stage the Stingray balloon can be withdrawn like an OTW balloon with the use of a “trap” balloon (Fig. **17**) to ensure stability of the distal wire position. The procedure is then completed in the standard manner with balloon angioplasty and stent insertion. 

Should re-entry be unsuccessful, repeat attempts can be made with the Stingray balloon in different positions by either rewiring and extending of the sub-intimal track with the CrossBoss catheter or by moving (“bob-sledding”) the deflated Stingray balloon proximally/distally within the sub-intimal space (usually on a wire).

## MANAGEMENT OF THE SUB-INTIMAL SPACE 

**Fig. (18):**
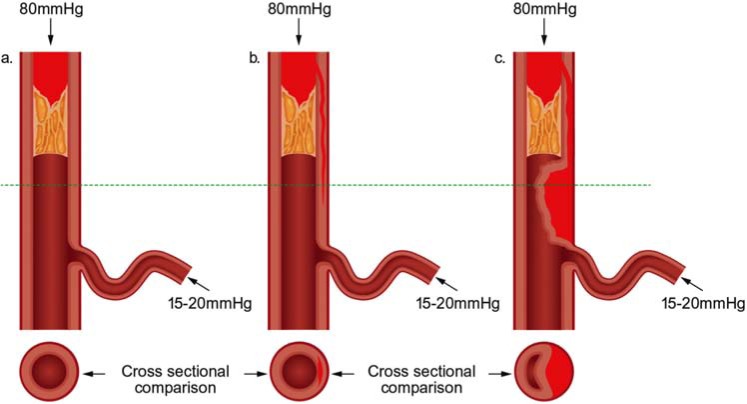
“The problem”.

Use of the subintimal space allows rapid negotiation of long occluded segments where ambiguity exists. The relative longitudinal and transverse weakness of this space is exploited in dissection re-entry strategies, but can predispose to large intramural haematoma formation as the sub-intimal space is exposed to arterial pressure (Fig. **18**). Haematoma formation within the sub-intimal space has two consequences in this setting: the effectiveness of the Stingray re-entry system is reduced as it is no longer closely apposed to the luminal wall when inflated, and loss of distal visualisation occurs due to compression of the true lumen by the haematoma. 

**Fig. (19):**
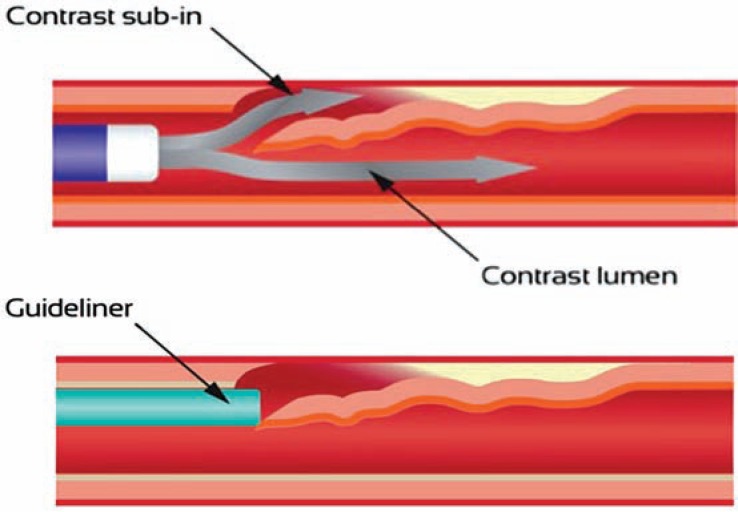
Upper panel = Hazards of antegrade contrast injections when working in the subintimal plane. Lower panel = Control of inflow with a Guideliner.

Risk factors for haematoma formation include large knuckle formation, poor control of wire position with distal propagation, increased levels of force required to cross the occlusion and antegrade contrast injections. Protection of the subintimal space can be achieved by the following measures: keeping the knuckle as small as possible; obsessive distal wire control when in the sub-intimal space during equipment exchanges to avoid inadvertent distal wire propagation, routine use of “trap” balloons (Fig. **17**), as well as “inflow management”. “Inflow management” can be achieved primarily by avoidance of antegrade injections until stent insertion (many operators remove the syringe from the antegrade manifold as a safeguard) but also by use of an OTW balloon or Guideliner catheter to control access to the subintimal space (Fig. **19**).

**Fig. (20):**
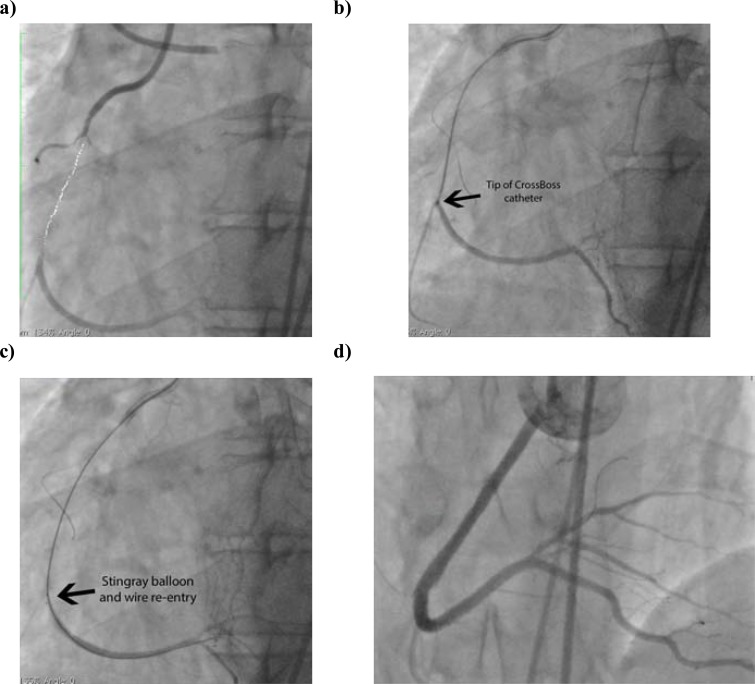
Case of successful CrossBoss and Stingray re-entry.

A good quality distal vessel, referred to as a “landing zone” increases the chances of successful re-entry, and, within the learning curve of the technique, operators should select CTOs with relatively large and disease free distal vessels (Fig. **20**). Use of a relatively disease free section for re-entry may, however, require extension of the dissection plane distal to the occlusion. It is important to note that some compromise may be required (between length of distal dissection and diseased vessel) to avoid excessive sub-intimal dissection so as to reduce the length of stent required and minimise side branch loss. 

**Fig. (21):**
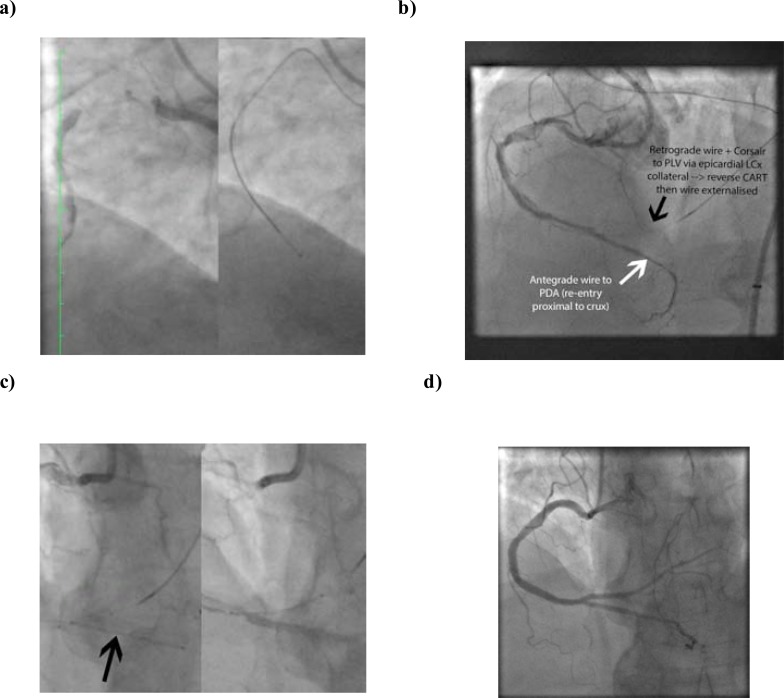
a. Long proximal RCA CTO and CrossBoss passage to distal vessel. b. Successful re-entry to distal vessel at crux with wire in distal PDA true lumen but no flow to PLV as origin dissected → successful retrograde wiring of PLV via epicardial LCx collateral and reverse CART in distal RCA to allow externalization of PLV retrograde wire c. Insertion of PDA then PLV stents (minicrush) with preservation of both branches. Excellent final result with TIMI 3 flow in both branches.

Case anatomy should determine which lesions are best treated by dissection re-entry; the presence of a bifurcation (of a major side branch) at the distal cap, for example, would argue for a primary retrograde approach (or “hybrid” approach in certain scenarios – see Fig. **21**). 

Evidence supporting the use of the Crossboss-Stingray system includes the FAST-CTO trial [33, 34] (US Facilitated Antegrade Steering Technique in Chronic Total Occlusions) and a European multicenter study [37]. In the FAST-CTO trial,

150 “refractory” CTOs (previous failed attempt or failed concurrent attempt) were attempted with the CrossBoss-Stingray system. The primary endpoint was successful placement of the guidewire within the distal true lumen (technical success), with secondary endpoints of fluoroscopy time, procedure time and MACE to 30 days. Exclusion criteria included vein grafts, in-stent occlusions, aorto-ostial occlusions, a large branch at the distal cap or a small distal vessel caliber (<1.5mm). The overall technical success rate was 77%. The CrossBoss crossed into the distal true lumen in 56 lesions and the Stingray balloon and wire facilitated distal true lumen re-entry in another 59 lesions. A learning curve was evident, and the second half of cases undertaken had a success rate of 86%. In addition, the 30 day MACE was low at 4.8% with five device related perforations, none of which required pericardiocentesis or surgery. Furthermore, average fluoroscopy time (44 mins) and procedure time (105 mins) were low and compared favorably to historical controls. 

## DELIVERY OF EQUIPMENT

**Fig. (22):**
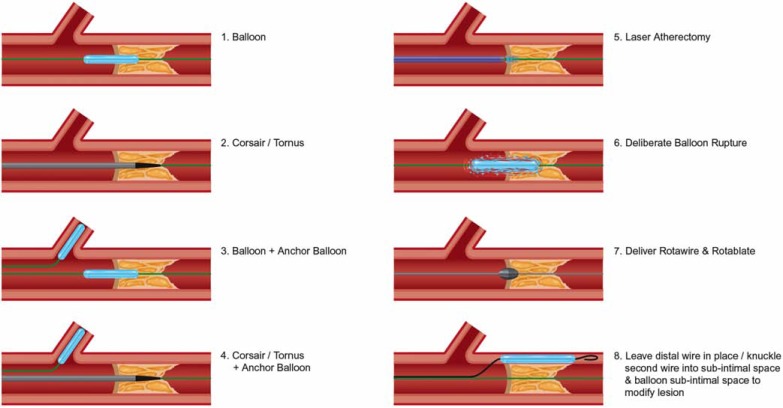
Algorithm for delivery of equipment once wire position is achieved in the distal vessel.

Once a stable wire position is achieved in the distal true lumen, the final challenge of antegrade CTO PCI is delivery of equipment over the wire, which can be difficult in the setting of calcification and tortuosity in particular. A step-wise algorithm for strategies to assist in delivery of equipment is presented in (Fig. **22**). 

## UNRESOLVED ISSUES

Although there have been major advances in techniques for CTO PCI, with high success rates having been achieved by expert operators in complex lesions, for this strategy to be regarded as truly successful, the high procedural success rates reported in expert operator’s hands, need to be replicated in a much broader group of operators. These techniques thus need to be readily teachable and reproducible.

Previously cited concerns regarding CTO PCI have included high procedure times and radiation exposure, as well as cost in terms of cathlab time and equipment use. For CTO PCI uptake to increase, procedures need to be efficient and cost effective. Antegrade subintimal dissection strategies allow long segments of occlusions to be negotiated rapidly and safely, with further refinements in re-entry techniques offering the potential for continued improvements in strategical efficiency. 

Although concerns linger regarding the long term impact of stent deployment in the sub-intimal space (whether this is via antegrade or retrograde dissection) no clinical signal has yet been identified in studies looking at differences between “intimal” and “sub-intimal” stenting [10]. Further reassurance would be provided by prospective studies addressing this issue. 

## CONCLUSIONS

Whilst made on the foundations provided by advances in technology and technique, the most fundamental progress seen in CTO PCI has been strategic. The “hybrid” approach is centered on a strategy determined by vessel anatomy but critically is not committed inflexibly to that strategy. Ideally, there exists a seamless interplay of antegrade wiring, antegrade dissection re-entry and retrograde approaches as dictated by procedural factors.

Antegrade wire escalation with intimal tracking remains the preferred initial strategy in short CTOs without proximal cap ambiguity. More complex CTOs, however, usually require either a retrograde or an antegrade dissection re-entry approach, or both as part of a hybrid CTO strategy.

Antegrade dissection re-entry is well suited to long occlusions where there is a good distal vessel and limited “interventional” collaterals. As direct antegrade wiring is unlikely to be successful or efficient, early use of a dissection re-entry strategy will increase success rates, reduce complications and minimize radiation, contrast use and procedure time. Antegrade dissection can be achieved with a knuckle wire technique or the CrossBoss catheter whilst re-entry will be achieved in the most reproducible and reliable fashion by the Stingray balloon/wire. As potential loss of side branches remains an issue, it should be avoided where a large side branch exists. It remains to be seen, however, whether use of newer dissection re-entry strategies will be associated with lower restenosis rates compared with the more extensive strategies such as STAR and whether stent insertion in the subintimal space is associated with higher rates of late stent malapposition and stent thrombosis. It is to be hoped that the algorithms, which have been developed to guide CTO operators, allow for a better transfer of knowledge and skills, which in turn should increase uptake and acceptance of CTO PCI as a whole.

## Figures and Tables

**Table 1.** Standard microcatheters in use.

	Finecross (Terumo)	Supercross (Vascular perspectives)	Valet (Volcano)	Corsair (Asahi)	Tornus (Asahi)
**Composition/design **	-Stainless steel braid structure -Hydrophilic coating for distal 60cm -PTFE inner layer	-Stainless steel braid structure -Hydrophilic coating for distal 40cm -PTFE inner layer -45°, 90°, 120° angled tip versions	-Stainless steel braid structure with variable metal wind thickness and powder plastic coating -Hydrophilic coating distal 30cm -Shapeable distal tip	-Tungsten braiding with 10 elliptical steel braids -Soft, tapered tip -Hydrophilic polymer coating for distal 60cm -Inner polymer lumen	-Eight individual stainless steel wires, braided with a left-handed thread
**Diameter/ length **	-Tapers from 2.6Fr to 1.8Fr over entire catheter -Available 130 and 150cm length	-Tapers from 2.5Fr to 1.8Fr -Available 130 and 150cm length	-Available 2 diameters (1.8Fr and 3.5Fr) - Available 130 and 150cm length	-2.8Fr with tapered distal tip (0.016 inch) -Available 135 (antegrade) and 150cm (retrograde) length	-Available in two sizes: Tornus (2.1Fr and 2.6Fr) -135cm length
**Instructions for use **	-Rotation in either direction facilitates forward motion	-Rotation in either direction facilitates forward motion	-Rotation in either direction facilitates forward motion	-Rapid rotation in either direction enhances forward motion	-Rotated into lesion counter-clockwise (maximum 20 turns in one go) and withdrawn clockwise -Should not be flushed (porous design and can splay wires)
**Indication **	-Provision of support for guidewire crossing	-Provision of support for guidewire crossing	-Provision of support for guidewire crossing	-Provision of support for guidewire crossing -Collateral channel crossing (especially retrograde)	-Highly supportive catheter -Allows balloon tracking (once distal wire position achieved when other microcatheters or OTW balloons will not cross) or affords rotawire exchange and therefore useful combination with rotablation

## ABBREVIATIONS

CTO: = chronic total occlusion
PCI: = percutaneous coronary intervention
CABG: = coronary artery bypass grafting
CCTA: = cardiovascular computed tomography angiography
LAD: = Left anterior descending
RCA: = right coronary artery
CART: = controlled antegrade and retrograde tracking
IVUS: = intravascular ultrasound
STAR: = sub-intimal tracking and re-entry
LAST: = limited antegrade subintimal tracking
OTW: = over the wire
